# Interference phase-contrast imaging technology without beam separation

**DOI:** 10.1038/s41598-018-38359-9

**Published:** 2019-02-11

**Authors:** Seiji Nishiwaki, Kenji Narumi, Tsuguhiro Korenaga

**Affiliations:** Technology Innovation Division, Panasonic Corporation, 3-1-1 Yagumo-nakamachi, Moriguchi City, Osaka 570-8501 Japan

## Abstract

Interferometers are widely used in science and industry to measure small displacements, changes in refractive index, and surface irregularities. In all interferometers, including phase-contrast microscopes and DICs (differential interference contrast microscopes), light from a single source is split into two beams that travel along different optical paths. They are then recombined to produce interference. The fundamental operation of beam separation makes device configuration more complex and adds to the bulk of the equipment. In this study we propose a new method of observing phase-contrast images without beam separation by using self-interference inside a grating coupler structure disposed on the observation plane. We experimentally demonstrate that the self-interference principle can generate phase-contrast images using a simple configuration. From measurements using a multilevel phase plate, we confirm its phase-contrast depth resolution to approach one- tenth of a wavelength.

## Introduction

Interferometers^1–13^ are widely used in science and industry to measure small displacements, changes in refractive index, and surface irregularities. In all interferometers, including phase-contrast microscopes^14–17^, DICs^18–21^ (differential interference contrast microscopes) and grating interferometers^22,23^, light from a single source is split into two beams that travel along different optical paths. They are then recombined to produce interference. Frits Zernike invented the phase-contrast microscope in the early 1930s by adding a phase plate to shift the phase of the reference beam to a lens-based microscope, which separates and then recombines two beams. He was awarded the Nobel Prize in 1953 for this achievement. However, images observed using phase-contrast microscopes were very dark, due to attenuation of the background light (the reference beam) used to increase the interference contrast, because the light value of the scattered light (sample beam) was far smaller than that of the background light. The DIC, developed by Georges Nomarski in 1952, overcame this problem by separating the light source into a P-polarized light and an S-polarized light using Wollaston prisms. These two beams are reference beams or sample beams relative to each other. Images observed using the DIC were substantially brighter because the light intensity of the sample beam matched that of the reference beam; however, this separation method of applying polarization generated side effects that made it unsuitable for measuring polarized samples.

Interference microscope technology, which has made a significant contribution to science and industry, is currently premised on beam separation, and its development has hitherto focused on how to separate the beam and how to recombine the separated beams.

## Operational Principles

**Figure 1:**
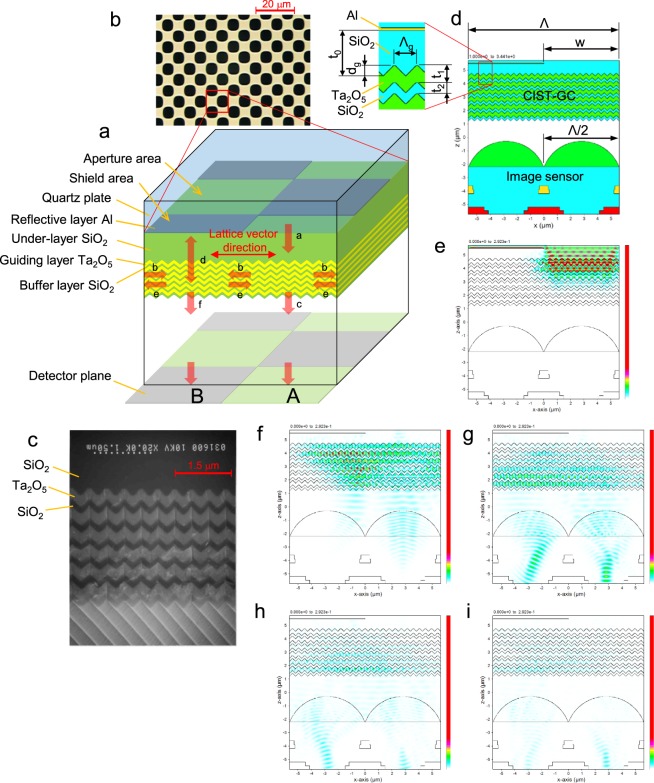
Configuration and operational principles. (**a**) Cross-sectional perspective view of the CIST-GC and propagation route of light. Vertically incident light **a** passing though the aperture area excites guided light **b** or goes straight down through the stacked layers as transmitted light **c**. Guided light **b** or **e** propagates to right and left along the lattice vector of the gratings while emitting radiated light vertically. After the radiated light mutually interferes under the shield area as shown by the arrow **d**, radiated light **f** is created. Transmitted light **c** is also interfered with by the radiative light under the aperture area. The transmitted light **c** and the radiated light **f** are detected by square detectors as light quantity A and B, respectively. (**b**) Microscopically observed plan view of the aluminum checker-pattern. The aperture shape of the aluminum pattern becomes elliptical due to the laterally etched effect. (**c**) Cross-sectional SEM photograph of the CIST-GC. The six paired layers of a guiding layer (Ta_2_O_5_) and a buffer layer (SiO_2_) are stacked while maintaining a triangular cross-section. (**d**) Cross-sectional chart of refractive index distribution when combining the CIST-GC with an image sensor. Film thickness: reflective layer (Al) = 0.05 µm, under-layer (SiO_2_) t_0_ = 0.850 µm, guiding layer (Ta_2_O_5_) t_1_ = 0.344 µm, buffer layer (SiO_2_) t_2_ = 0.220 µm. Grating shape: pitch Λ_g_ = 0.45 µm, depth d_g_ = 0.20 µm. Al checker pattern: pitch Λ = 11.2 µm, aperture width w = 5.6 µm. Image sensor: pixel width Λ/2 = 5.6 µm. (**e**–**i**) Time-series charts of cross-sectional light intensity distribution simulated by FDTD. The boundary condition is the PML (perfect matched layer). Light source: wavelength λ = 850 nm, pulse width = 10 µm. Time interval: 9.6 µm. The pulse light is separated to right and left in the guiding layers and is detected ultimately by the sensors beneath the aperture area and the shield area.

We have developed a new imaging method that we term CIST (contrast image sensing technology) by combining a grating coupler structure (CIST-GC) with an image sensor. Figure 1a shows a cross-sectional perspective view of the CIST-GC and the light propagation route.

The CIST-GC is constructed as follows. An aluminum film (reflective layer, 0.050 µm thick) is formed on a quartz substrate (0.5 mm thick) and is fully etched with a checkered pattern (pitch Λ = 11.2 µm) using a wet-etching process. Figure 1b shows a plane-view micrograph of the reflective layer. After a silicon oxide film (under-layer, thickness t_0_ = 0.850 µm) is formed on the aluminum layer, linear gratings (pitch Λ_g_ = 0.45 µm, depth d_g_ = 0.20 µm) are etched onto the surface of the under-layer by electron beam lithography. A paired layer comprising a tantalum oxide film (guiding layer, thickness t_1_ = 0.344 µm) and a silicon oxide film (buffer layer, thickness t_2_ = 0.220 µm) are formed six times on the linear gratings using a self-healing effect in an auto-cloning process^24^. These six paired layers are formed while maintaining a triangular cross-section. Figure 1c shows a cross-sectional SEM photograph of the CIST-GC. The tantalum oxide layers with gratings work as a grating coupler^25,26^ for vertically incident light.

A light beam from a laser source penetrates a sample and reaches the CIST-GC without beam separation. In Fig. 1a, the incident light **a** into the CIST-GC penetrates the aperture area of the reflective pattern and excites guided light **b** to right and left along the lattice vector of the gratings, while the rest penetrates the paired layers as transmitted light **c**. The guided light **b** of TE (transverse electric) or TM (transverse magnetic) mode is individually excited by the incident light a of S or P polarization. The guided light **b** travels between the guiding layers because the guiding layers are closely stacked, and becomes guided light **e** after passing under the shield area of the reflective pattern. Guided light beams aligned in the same direction interfere with each other. Guided light **b** or **e** travels while emitting radiative light vertically, and a part of the radiative light creates new guided light. After the radiative light mutually interferes under the shield area shown by the arrow **d**, radiated light **f** is created. Transmitted light **c** is also interfered with by the radiative light under the aperture area. We call these forms of interference inside the CIST-GC ‘self-interference’. The transmitted light **c** and the radiated light **f** are detected by square detectors of width Λ/2 as light quantities A and B, respectively. Obviously, phase differences of the incident light between the two aperture areas sandwiching one shield area along the lattice vector direction will effect A and B.

Figure 1d shows a cross-sectional chart of refractive index distribution combining the CIST-GC with an image sensor. Figure 1e–i show time-series charts of cross-sectional light intensity distribution simulated by the FDTD^27^ (finite differential time domain). As time elapses, the incident light of wavelength λ = 850 nm and pulse width 10 µm is separated to right and left in the guiding layers, and is detected ultimately by the sensors beneath the aperture area and the shield area.

## Arithmetic Processing and Optical Characteristics of the CIST-GC

**Figure 2:**
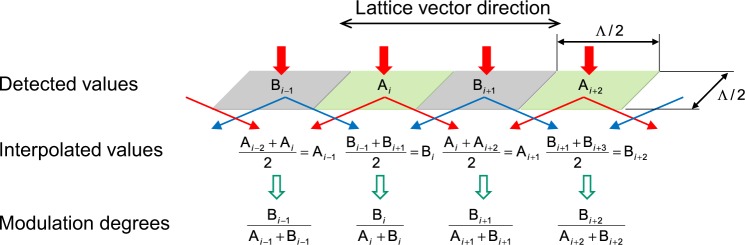
Arithmetic processing. The detected image is divided into a uniform mesh, and detected values are measured in their respective squares. Interpolated values are calculated from the alternate detected values. The MD (modulation degree) is calculated for all squares from the detected value and the interpolated value located at the same place.

Figure 2 shows the arithmetic processing employed to derive the MD (modulation degree): The detected image is divided into a uniform mesh of width Λ/2 aligned on the checker pattern. Detected values like A_i_ and B_i+1_ are measured at respective squares. Interpolated values like A_i+1_ and B_i_ are calculated along the lattice vector direction from the alternate detected values. The MD is calculated for all squares from the detected value and the interpolated value located at the same place.

**Figure 3:**
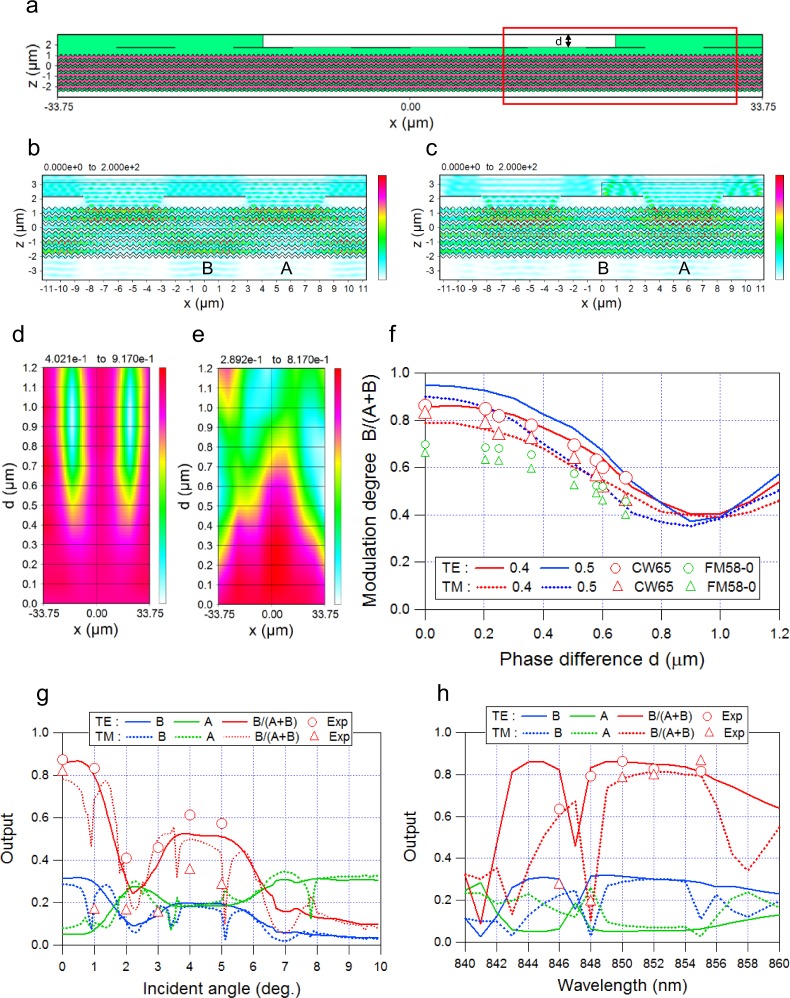
Optical characteristics. (**a**) Cross-sectional chart of refractive index distribution in a 2D analytical model under PBC. Two reciprocal phase steps of height d are placed on the center of the shield areas at an even distance. (**b,c**) Charts of cross-sectional light intensity distribution for d = 0.0 µm and d = 0.94 µm. The analytical region is confined to the area surrounded by the red border in (a). The CW light source is used under PBC. (**d,e)** Contour plots of the MD. The detector position x on the abscissa and the phase difference d on the ordinate for TE mode and TM mode, respectively. (**f**) Phase-difference dependency of the MD. The analytical area is shown in (a). The aperture ratio (w/Λ) is set as a parameter. Markers show measurement results. (**g,h**) Output characteristics for incident angle and wavelength. Outputs on the vertical axis are light quantity A, B normalized by incident light quantity, and the MD B/(A + B). Markers show measurement results of the MD.

Figure 3a shows a cross-sectional chart of refractive index distribution in a two-dimensional (2D) analytical model: the analytical width is close to six checker periods (11.25 * 6 = 67.5 µm, with multiple numbers of grating pitches Λ_g_), and two reciprocal phase steps of height d placed at the center of the shield areas at an even distance (67.5/2 = 33.75 µm).

If the analytical region is confined to the area surrounded by the red border in Fig. 3a, and a CW (constant wave) source is used under the PBC (period boundary condition), cross-sectional light intensity distributions for d = 0.0 µm and d = 0.94 µm are computable by FDTD, as shown in Fig. 3b,c. Comparing Fig. 3c to Fig. 3b, the light quantity A is greater than B in Fig. 3c, while B is greater than A in Fig. 3b. This means that allocation to A and B depends on the presence or absence of the phase step.

Figure 3d,e show a contour plot of the MD with the detector position x on the abscissa and the phase difference d on the ordinate for the TE mode and TM mode, respectively. The detector position corresponds to the twelve positions under the aperture areas and the shield areas as shown in Fig. 3a. In TE mode, the MD falls to a valley-shaped low, but only directly beneath the phase steps. On the other hand, the valley for the TM mode spreads across several detector positions. This is explained by differences in coupling factors (i.e., radiation decay factors)^28^ between the TE mode and the TM mode. In short, the TM mode, which has a smaller decay factor, is more transmissive and is more likely to cause interference than the TE mode.

Figure 3f shows the phase difference dependency of the MD, calculated using modified RCWA^29,30^ (rigorous coupled wave analysis; see Methods), for which the analytical area is shown in Fig. 3a. The MD is defined by the value just below the phase step located on the shield area. The aperture ratio (w/Λ) is set as a parameter in consideration of the elliptical aperture shape shown in Fig. 1b. From Fig. 3f, the MD curves for TE and TM mode are sinusoidal, with a peak at d = 0 µm and a bottom at d ≈ 0.94 µm (corresponding to a phase shift of π).

Output characteristics putting the incident angle (at λ = 850 nm) and a wavelength on the horizontal axis are simulated in Fig. 3g,h, respectively. Outputs on the vertical axis are light quantity A and B, normalized by incident light quantity, and the MD B/(A + B). In TE mode, the MD maintains a high level of over 0.8 in the range of angle deviation ±1.0 degree and wavelength deviation from 848–855 nm. Measurements of the MD were also plotted as markers in Fig. 3g,h (see Methods): they closely coincided with the analytical results.

## Experimental Methods and Results

**Figure 4:**
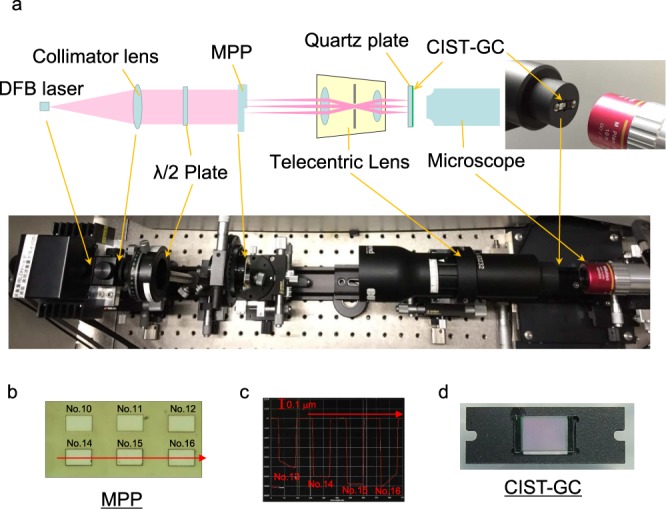
Experimental frame for measurement of phase-difference dependency. (**a**) Total configuration picture for measurement of phase-difference dependency. The light beam from the laser source reaches the CIST-GC without beam separation. (**b**) Partial appearance picture of the MPP. Several flat dents with different depths are formed by FIB. (**c**) Measured results of phase differences of the MPP. The dents Nos 13 and 16 are formed with inclined bottoms, while dents Nos. 14 and 15 are formed with a flat bottom. (**d**) The CIST-GC on a holder.

Figure 4a shows an experimental frame for measurement of phase difference dependency. After collimating light from a DFB (distributed feedback) laser source (Eagleyard, EYP-0852-DFB) whose emission wavelength is locked stably to close to 850 nm by a Peltier control, and after rotating the plane of polarized light using a half-wavelength plate, the light beam is incident vertically to an MPP (multilevel phase plate made from a quartz plate) on whose surface several flat dents are formed by FIB (focused ion beam technology). A part of the MPP surface is shown in Fig. 4b and the boundaries of the dents become phase differences with different depths, as measured by a stylus profiler (KLA-Tencor, P-10) shown in Fig. 4c.

Figure 4d shows a CIST-GC on a holder. The light beam penetrating the MPP forms an image onto the CIST-GC by means of a telecentric lens (Edmund, #63-232, magnification 1.7). The light distribution immediately after transmitting the CIST-GC is observed using a microscope (Edmund, #46-403 and #58-310) in place of an image sensor and the MDs are calculated from the detected electric image. It should be noted that the light beam from the laser source reaches the CIST-GC without beam separation.

**Figure 5:**
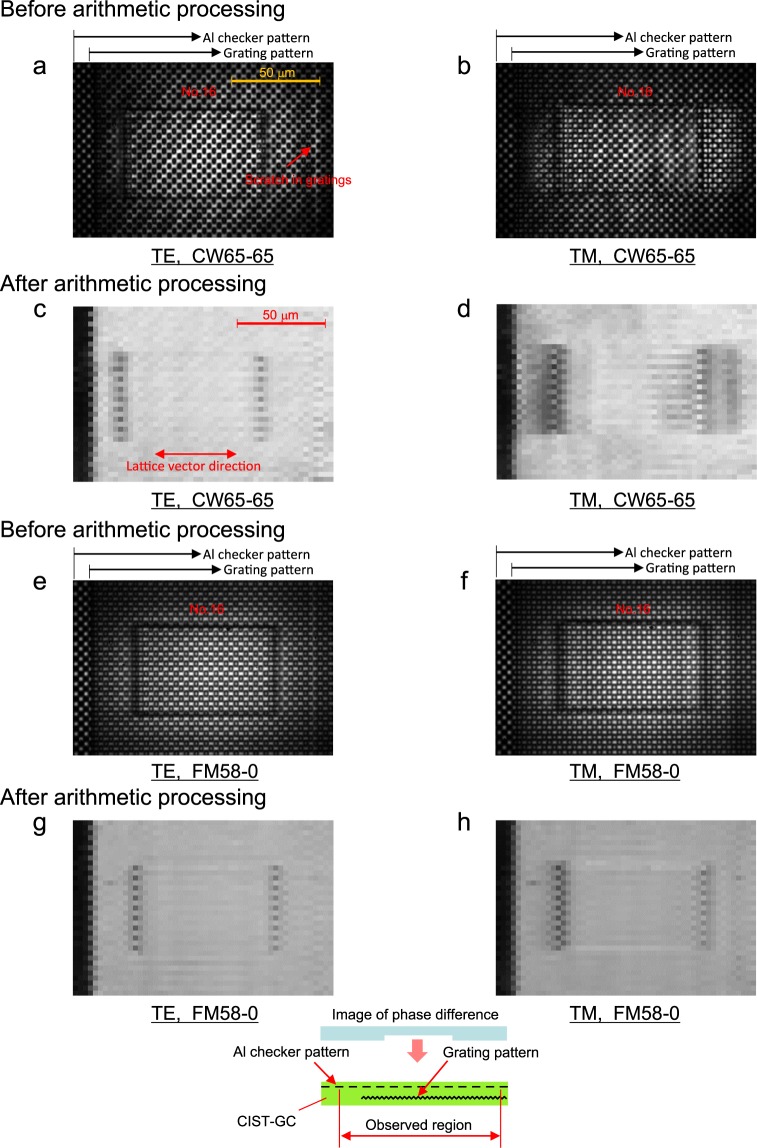
Images before and after arithmetic processing. (**a,b**) Observed raw images before arithmetic processing for the TE mode and TM mode. The light source is a CW emission of “CW65.” (**c,d**) Images of the MD after arithmetic processing for the TE mode and TM mode. The light source is a CW emission of “CW65.” The image of the MD darkens alternately along the phase difference line in a direction perpendicular to the lattice vector. (**e,f)** Observed raw images before arithmetic processing for the TE mode and TM mode. The light source is driven by the 600-MHz modulation signal in the range of 58 mA to 0 mA. The speckle interference is removed. (**g,h**) Images of the MD after arithmetic processing for the TE mode and TM mode. The light source is driven by the 600-MHz modulation signal in the range of 58 mA to 0 mA. The rough brightness is improved and the dark region of TM mode becomes narrower than that in (d).

Figure 5a,b (or 5e and 5f) show observed raw images before arithmetic processing for the TE mode and TM mode, respectively. Figure 5c,d (or 5g and 5h), respectively, show images of the MD after arithmetic processing in the TE mode and TM mode. The detection of phase-contrast is limited to cases in which a phase difference exists between two aperture areas sandwiching the shield area along the lattice vector. Therefore, in Fig. 5c,d, the image of the MD darkens alternately along the phase difference line that is perpendicular to the lattice vector. Measurements of the MD are plotted as markers of “CW65” in Fig. 3f. They accord closely with the analytical results for when w/Λ = 0.4. The phase-contrast depth resolution is expected to approach λ/10 (=(n-1)d, where d = 0.2 µm and n is the refractive index [=1.45] of the MPP), because all of the experimental MDs for d = 0.2 µm are smaller than those for d = 0.0 µm, as shown in Fig. 3f.

Since Fig. 5a,b include slanting bright and dark fringes caused by speckle interference in the laser light, there is a slight roughness in the bright region of Fig. 5c,d. Moreover, as shown in Fig. 5d, the dark region around the phase difference line becomes wider than that in Fig. 5c. This agrees with the results shown in Fig. 3d,e. These problems are resolved by deteriorating coherency of light (see Supplementary Section A).

Figure 5e–h show the results of superimposing a modulation signal of 600 MHz in the current range of 58 mA to 0 mA to deteriorate coherency, and respectively correspond to Fig. 5a–d. The speckle interference is eliminated in Fig. 5e,f, and rough brightness is also improved in Fig. 5g,h. The dark region of the TM mode in Fig. 5h becomes narrower than that in Fig. 5d, and closely approaches that of the TE mode in Fig. 5g. Measurements of the MD are also plotted as markers of “FM58-0” in Fig. 3f. They are equivalent to about 80% of the results for “CW65”.

On the premise of deteriorating coherency of light or when using LED light, the plane resolution of CIST is determined by half of the period of the reflective pattern after the interpolation effect of the arithmetic processing shown in Fig. 2. The aperture width, which is half of the period, needs a grating consisting of 4 lines or more (12–13 lines in our case) to excite the guided light (see Supplementary Section B). The grating period is determined by the wavelength divided by the effective index of the waveguide, making it almost half the wavelength. As a result, the plane resolution is improvable up to twice the wavelength.

## Conclusion

In conclusion, our results demonstrate that the CIST can detect phase-contrast images using a novel phase shift first derivative imaging technique based on the self-interference principle, and that it can achieve quantification at a phase-contrast depth resolution that approaches λ/10. Since the CIST detects phase differences between two aperture areas sandwiching a shield area, measurements become differential in the detection plane. This is similar to the sensing performance of the DIC (see Supplementary Section D), but there is no problem with polarization disturbance in the DIC. The telecentric lens is used here as an imaging lens, but it is only necessary if magnification of images is required (see Supplementary Section C). Unlike all other interference microscopy techniques, in which light beams are separated into two on the way to the imaging plain, the CIST causes phase-contrast interference with a single beam.

We believe that the CIST shows promise as a new method for creating next-generation phase-contrast imaging. We plan next to examine the combination of the CIST-GC with an image sensor, with the aim of expediting its practical use.

## Methods

### Simulation using the modified RCWA

Since light continues propagating along the guiding layers in the CIST-GC for a while, when using FDTD under a periodic boundary condition, it takes time for analytical variation to move toward equilibrium. Moreover, the computation load becomes very heavy during the simulation for phase difference dependency, because, in the simulation of phase differences, an analytical region exceeding 67.5 µm (i.e., a distance of over 33.75 µm between the two phase steps) is required to match the analytical results to the experimental results. Thus, RCWA, which is an analytical tool for standing waves, is selected over FDTD. RCWA is self-built here, because there is no commercially available software for analyzing the light values of detectors.

### Measurement of incident angle or wavelength dependency

The incident angle or wavelength dependency shown in Fig. 3g,h is measured without the MPP and the telecentric lens from the framework mentioned in Fig. 4a. To make incident angle dependency measurements, the optical system is combined with a tilting system. For wavelength dependency measurements, a Fabry-Pérot laser source (Sheaumann Laser, M9-852-0100-S5P), controlled by a Peltier system, is applied.

## Supplementary information

Supplementary Information to Interference phase-contrast imaging technology without beam separation
