# Supplementary material for: Interference phase-contrast imaging technology without beam separation
Source: Sci Rep. 2019 Feb 11;9:1753. doi: 10.1038/s41598-018-38359-9 (PMC6370786; doi:10.1038/s41598-018-38359-9)
Supplement: Supplementary file 1 — Supplementary Information to Interference phase-contrast imaging technology without beam separation [file 41598_2018_38359_MOESM1_ESM.pdf]

Supplementary Information to
Interference phase-contrast imaging technology without beam separation

Seiji Nishiwaki, Kenji Narumi, and Tsuguhiro Korenaga

Technology Innovation Division, Panasonic Corporation, 3-1-1 Yagumo-nakamachi, Moriguchi City, Osaka 570-8501, Japan

Supplementary section A

Control of coherence length in a DFB laser

It is well known [1-2] that the superimposition of a high-frequency wave on Fabry-Pérot lasers puts the oscillation spectrum in multimode oscillation, and that it reduces the speckle noise while shortening the coherence length. On the other hand, while maintaining single-mode oscillation, superimposition on the DFB laser can control the coherence length in the following way. Using an interferometer as shown in Figure S1a, we measured the relationship between the visibility [3] of interfering light and the difference in optical path length as a parameter under the laser driving conditions shown in Fig. S1b. Under the condition of a frequency modulation of 600 MHz and duty factor 50%, the four conditions for the peak and valley were selected. Coherence lengths can be estimated from the half-value difference of optical path length, as shown in Table 1Sc. Since the threshold current of the laser oscillation is 30 mA, the condition of "CW25-25" corresponds to an LED mode oscillation.

Supplementary section B

Spatial resolution of CIST

The phase difference dependencies of the MD for $w/\Lambda = 0.5$, calculated using modified RCWA, are shown in Figure S2a, parameterizing the reflective pattern period Λ under the same analytical model as Figure 3a. The variation widths of the MD, Δ(MD)s, are estimated from Figure S2a, and the relationship between Δ(MD) and $\Lambda/2$ is shown in Figure S2b. The curve of Δ(MD) has a maximum at $\Lambda/2 = 2.8125$ µm (or $\Lambda/2\lambda = 3.3$ at $\lambda = 850$ nm) and maintains a high level of over 0.6 in the range over $\Lambda/2 = 1.8$ µm (or $\Lambda/2\lambda = 2$, corresponding to 4 grating lines). Therefore, the spatial resolution of the CIST is estimated to be up to two times of the wavelength.

Supplementary section C

Observation of phase-contrast images in a lens-free model

Figure S3a shows an experimental framework for observation of phase-contrast images in a lens-free model. After collimating the light from the DFB laser source and after rotating the plane of polarized light using a half-wavelength plate, the light beam is vertically incident to the MPP. The surface of the CIST-GC is located face to face just behind the MPP surface. This CIST-GC is different from that shown in Fig. 1a, and the reflective layer of a stripe pattern (pitch Λ = 11.2 μm), the under-layer, and the paired layers are formed in reverse order on a quartz plate as shown Fig. S3b. The light distribution immediately after transmitting the CIST-GC is observed using a microscope, and the MDs are calculated from the detected electric image.

Figures S4a and S4b (or S4e and S4f), respectively, show observed raw images before arithmetic processing for the TE mode and TM mode. Figures S4c and S4d (or S4g an S4h) are, respectively, images of the MD after arithmetic processing for the TE mode and TM mode.

In Figures S4c and d, the image of the MD darkens along the phase difference line perpendicular to the lattice vector. If the shield area is aligned in the phase difference line, the dark region of the MD becomes straight in a seamless manner. However, if the phase difference line deviates from the shield area, the image of the MD becomes lighter. This makes it more difficult to adjust the location of the CIST-GC than for the checker shield pattern shown in Fig. 1b.

Since the images in Figs. S4a and S4b include speckle interference in the laser light, a slight roughness exists in the bright region of Figs. S4c and S4d. As shown in Fig.

S4d, the dark region around the phase difference line becomes wider than that in Figure S4c.

Figures S4e, f, g and h are the results obtained by superimposing a modulation signal of 600 MHz over the current range of 58 mA to 0 mA, and respectively correspond to Figs. S4a, b, c and d. The speckle interference is removed in Figs. S4e and S4f, and the bright roughness is also improved in Figures S4g or S4h. The dark region of the TM mode in Fig. S4h becomes narrower than that in Fig. S4d, and closely approaches that of the TE mode in Fig. S4g.

Supplementary section D

Difference between a DIC and a CIST

In a DIC, the two wave-fronts shift by δ along the x-axis as phase distributions $f_p(x)$ and $f_S(x)$ as shown in Figure S5a. After interfering, they create a wave-front of phase difference distribution $f_p(x)$-$f_s(x)$. This distribution is inverted in those parts corresponding to phase steps A and B. If the phase difference is less than π , one becomes dark and the other becomes bright. Figure S5g is an imaging result using a DIC microscope (DSX-510, Olympus) for the MPP (No. 12) aligned to the direction of the δ-shift, i.e., 45 degrees of rotation. The step images for A and B in Figure S5g include an inverted light-dark cycle, because the phase difference is over π, whereas the remaining two step images perpendicular to the direction of steps A and B are inconspicuous. Since the two wave-fronts $f_p(x)$ and $f_S(x)$ are not congruent due to the tilt between the optical axes of the separated light, the heights of phase steps are also different. The bright levels for the flat areas C and D in Figure S5g are therefore different, and these levels are indefinite and dependent on the phase differences. For the sloping steps shown in Figure S5b or S5c, the interfering wave-front takes the

form of a slope to the extent that δ and the light-dark changes are also sloping there. As shown in Figure S5h (an imaging result for a blazed grating of 70 lines/mm and 1.24 μm depth, Edmund #46-067), a great deal of contrast appears around the step regions, while light-dark changes caused by the incongruence between the wave-fronts are gentle around the sloping region (the extent of a shown in Figure S5c). These accord closely with the results of Figure S5c. However, there might be a difference in vision such as interference color since the DIC uses a white-light LED as its illumination source.

In the CIST, the MD distribution $g(x_i)$ is obtained discretely from the effective phase difference between the Λ-interval points $f(x_{i-1})$ and $f(x_{i+1})$ on the phase distribution $f(x)$ as shown in in Figure S5d. The MD distributions corresponding to steps A and B are dark together, unlike the DIC if the phase step is less than π, and the MDs corresponding to the flat area are uniformly close to the same level (i.e., 1.0), unlike a DIC. For the sloping steps shown in Figure S5e or S5f, the MD distribution adopts a discrete slope-shape to the extent of the slope of the phase. The light-dark change is also sloped, as shown in Figure S5i (an imaging result of TE mode and FM58-0 after arithmetic processing using a telecentric lens of magnification 1.7 for the above blazed grating), whereas moiré stripes appear due to a mismatch between the period (Λ=11.2 μm) of the CIST reflective pattern and the blazed grating period (1000/70*1.7 = 24.3 μm) magnified by the telecentric lens. Since the MD distribution is detected discretely, a steep and narrow slope cannot be projected as a slope shape.

The spatial resolution of CIST is determined by $\Lambda/2$ (half of the period of the reflective pattern). In the same way, the shift amount δ in DIC affects the spatial resolution of DIC, because it feathers the step boundary of the wave-front.

References

(1) Tarucha, S., Otsuka, K. Response of semiconductor laser deep sinusoidal injection current modulation. *IEEE Journal of Quantum Electronics*,Vol. QE-17, No. 5, 810-816 (1981).

(2) Russer, P. Direct modulation of semiconductor injection lasers. *IEEE Trans. Micro. Theory and Tech.*,Vol. 30, No. 11, 1809-1821 (1982).

(3) Born, M. and Wolf, E. *Principle of Optics* (Pergamon Press, 1965).

a

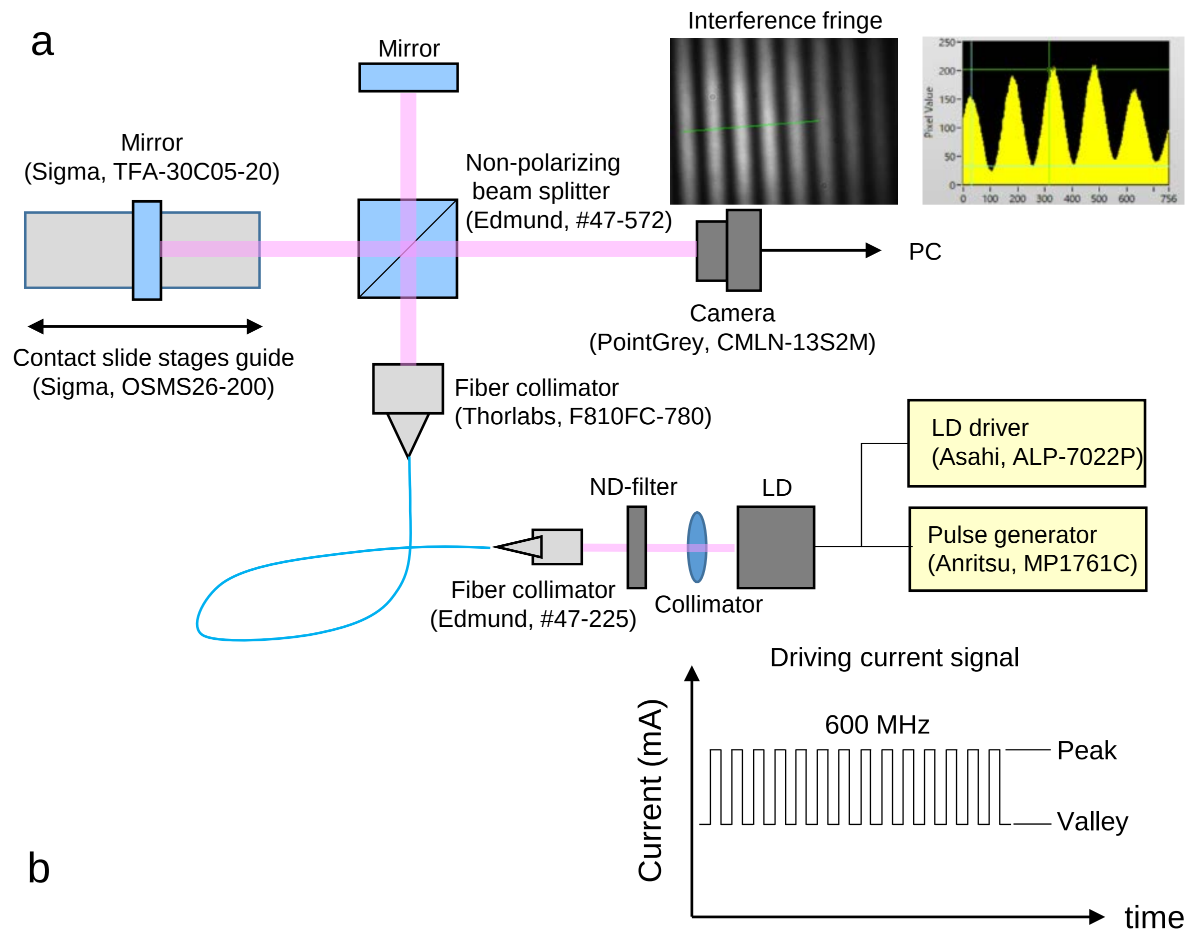

b

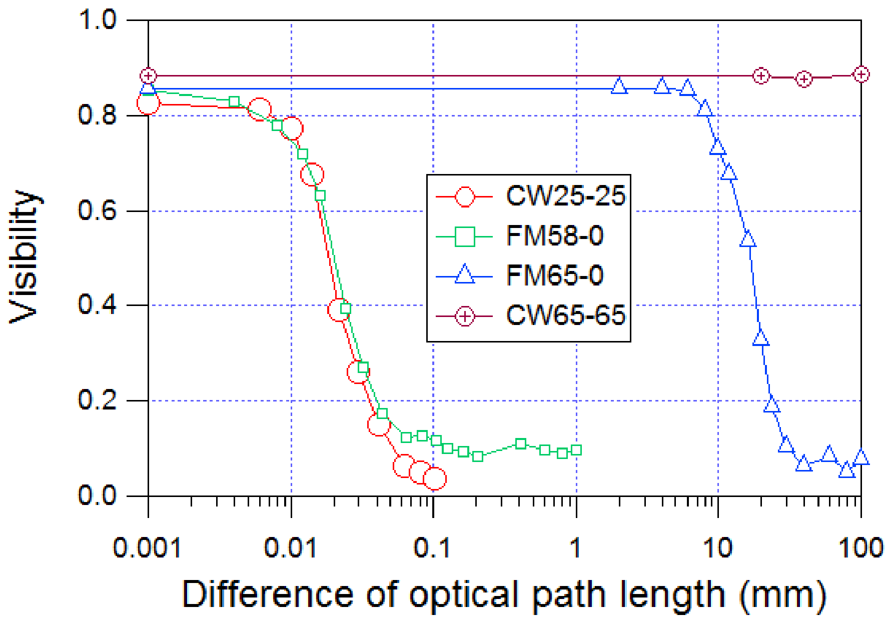

c

Driving condition Peak-Valley (mA)	Coherence length (mm)
CW 25-25	0.032
FM 58-0	0.036
FM 65-0	25
CW 65-65	>1000

<u>Figure and Table S1.   Driving condition and coherence length</u>

a

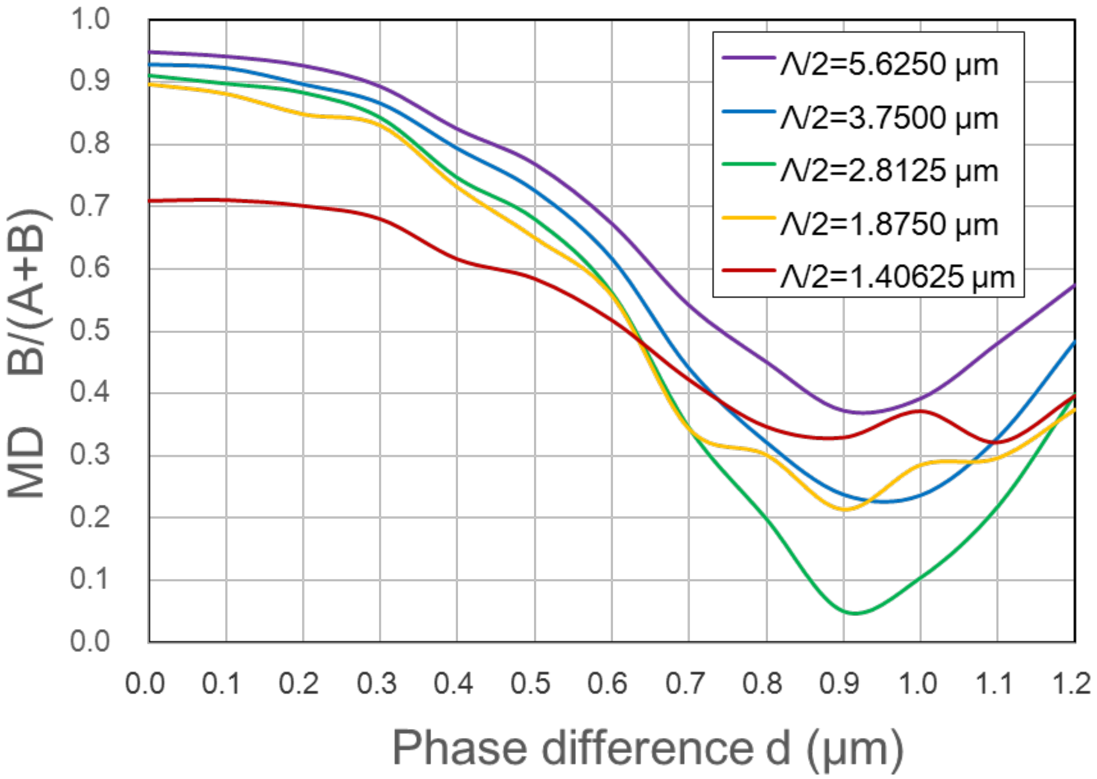

b

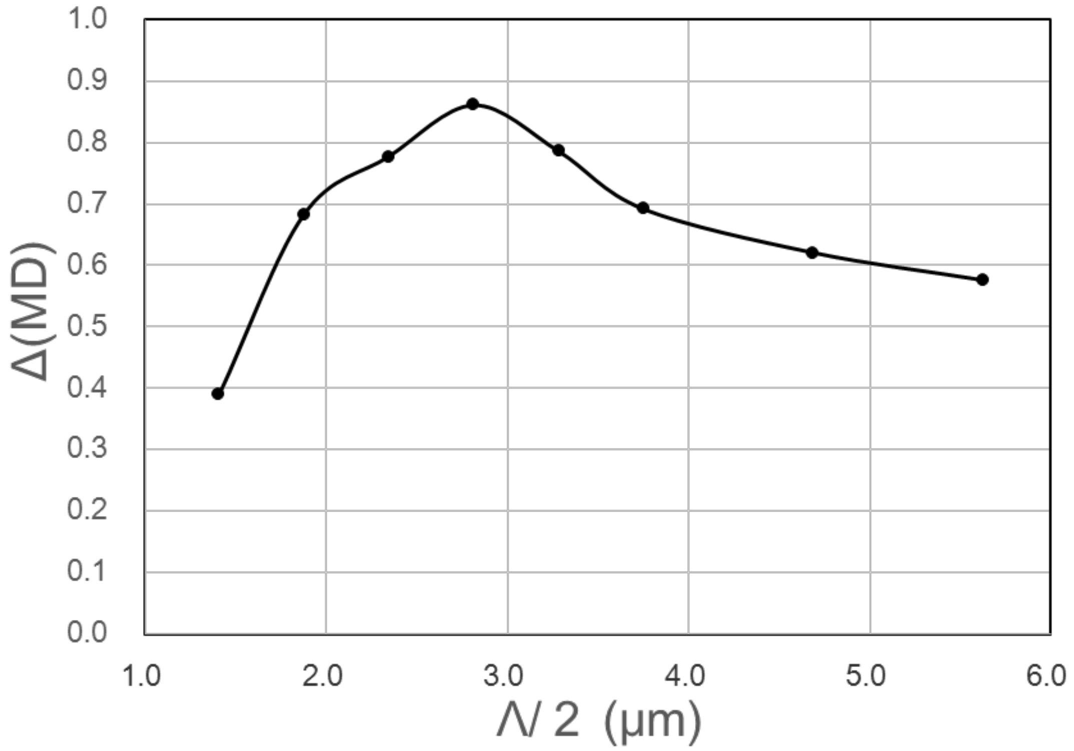

<u>Figure S2. Spatial resolution of CIST</u>

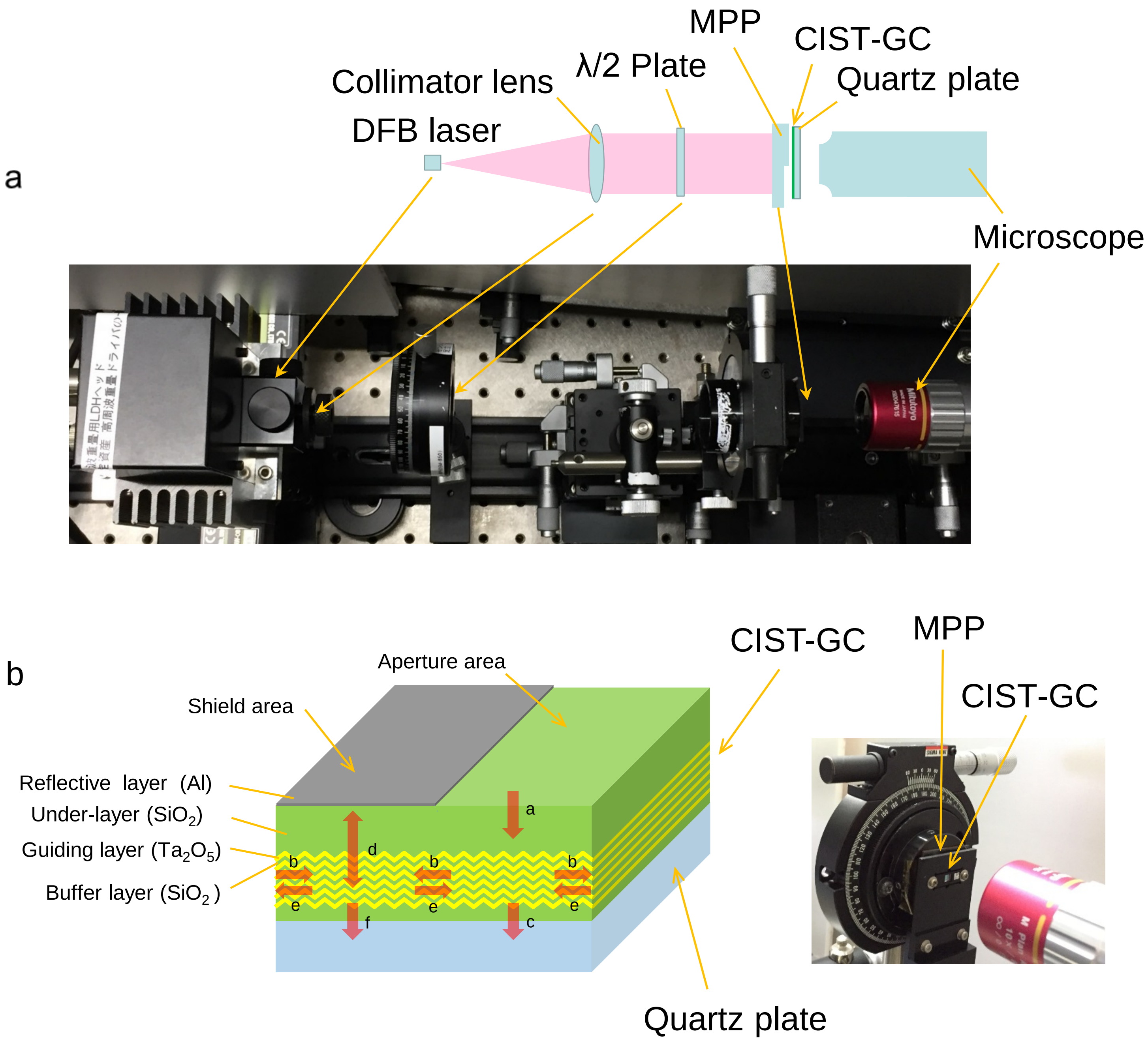

<u>Figure S3. Experimental frame for observation of phase-contrast images in a lens-free model</u>

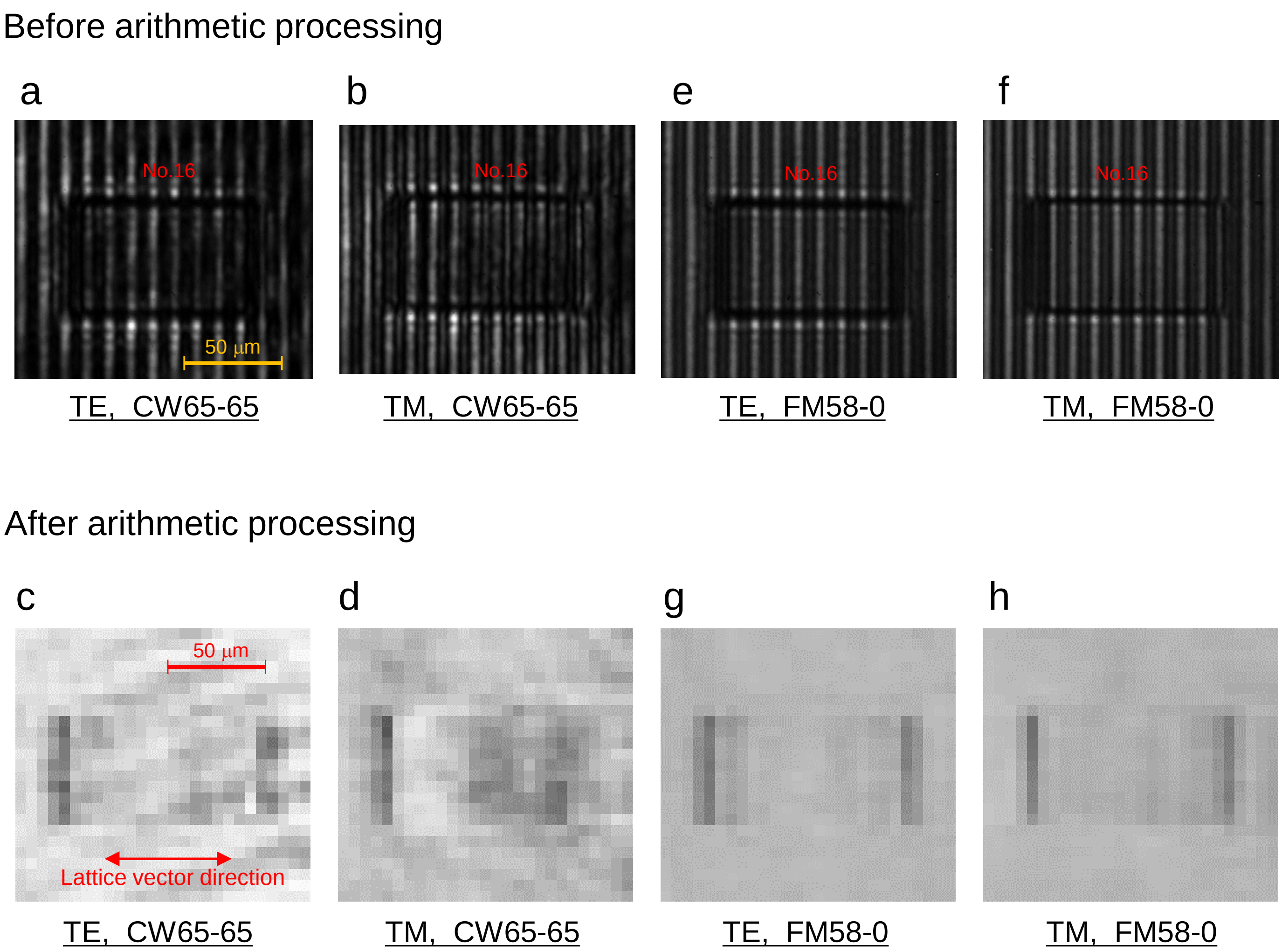

Figure S4. Images before and after arithmetic processing in the lens-free model

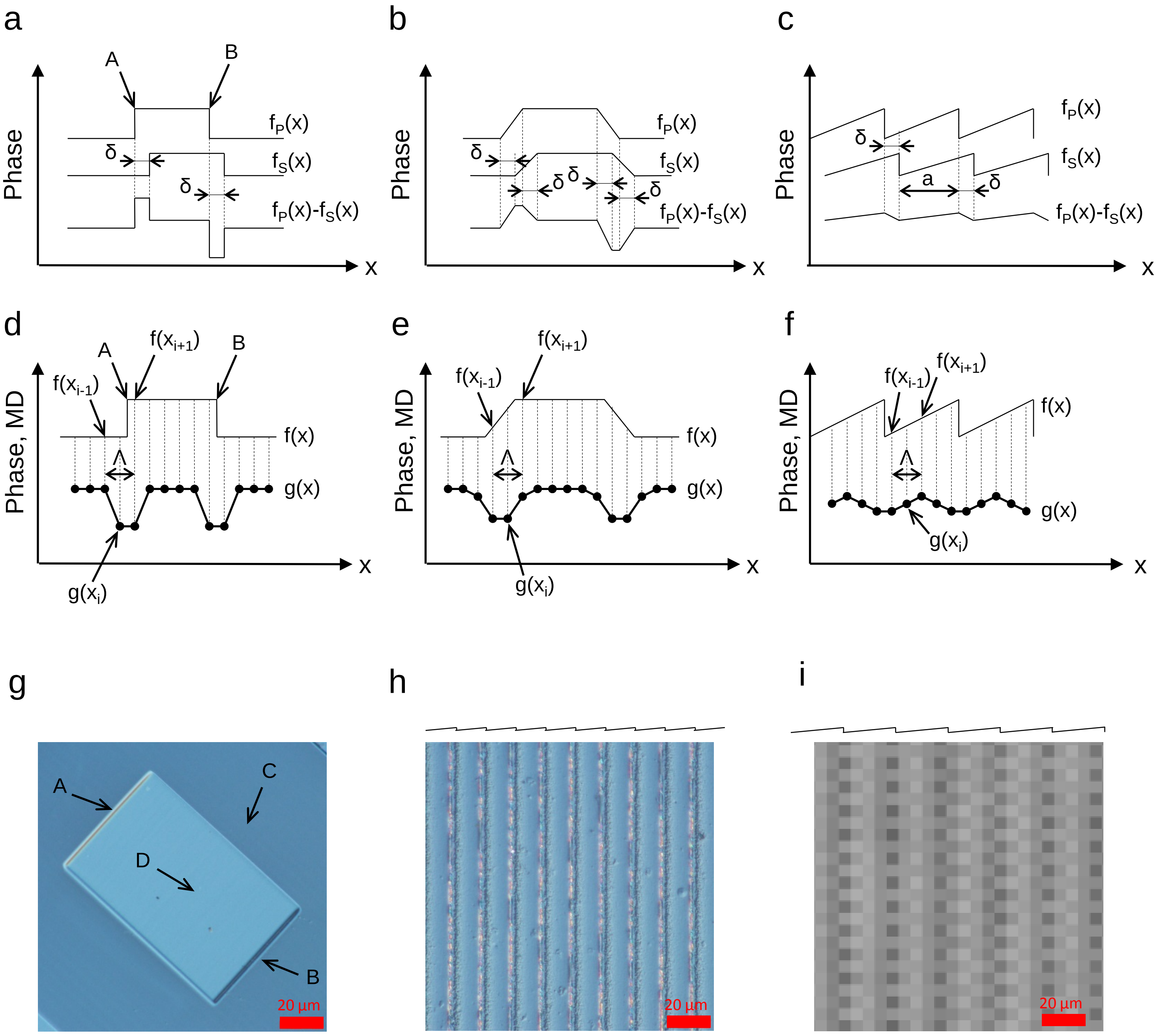

Figure S5. Difference between DIC and CIST
